# Proteinase-Activated Receptor-1 and Immunomodulatory Effects of a PAR1-Activating Peptide in a Mouse Model of Prostatitis

**DOI:** 10.1155/2013/748395

**Published:** 2013-12-29

**Authors:** M. Mark Stanton, Lisa K. Nelson, Hallgrimur Benediktsson, Morley D. Hollenberg, Andre G. Buret, Howard Ceri

**Affiliations:** ^1^Department of Biological Sciences, University of Calgary, 2500 University Drive NW, Calgary, AB, Canada T2N 1N4; ^2^Inflammation Research Network, University of Calgary, 2500 University Drive NW, Calgary, AB, Canada T2N 1N4; ^3^Biofilm Research Group, University of Calgary, 2500 University Drive NW, Calgary, AB, Canada T2N 1N4; ^4^Department of Pathology and Laboratory Medicine, Calgary Laboratory Services, Foothills Medical Centre, 1403 29 Street NW, Calgary, AB, Canada T2N 2T9; ^5^Department of Physiology and Pharmacology, University of Calgary Health Sciences Center, 3330 Hospital Drive NW, Calgary, AB, Canada T2N 4N1

## Abstract

*Background*. Nonbacterial prostatitis has no established etiology. We hypothesized that proteinase-activated receptor-1 (PAR1) can play a role in prostatitis. We therefore investigated the effects of PAR1 stimulation in the context of a new model of murine nonbacterial prostatitis. *Methods*. Using a hapten (ethanol-dinitrobenzene sulfonic acid- (DNBS-)) induced prostatitis model with both wild-type and PAR1-null mice, we examined (1) the location of PAR1 in the mouse prostate and (2) the impact of a PAR1-activating peptide (TFLLR-NH_2_: PAR1-TF) on ethanol-DNBS-induced inflammation. *Results*. Ethanol-DNBS-induced inflammation was maximal at 2 days. In the tissue, PAR1 was expressed predominantly along the apical acini of prostatic epithelium. Although PAR1-TF on its own did not cause inflammation, its coadministration with ethanol-DNBS reduced all indices of acute prostatitis. Further, PAR1-TF administration doubled the prostatic production of interleukin-10 (IL-10) compared with ethanol-DNBS treatment alone. This enhanced IL-10 was not observed in PAR1-null mice and was not caused by the reverse-sequence receptor-inactive peptide, RLLFT-NH_2_. Surprisingly, PAR1-TF, also diminished ethanol-DNBS-induced inflammation in PAR1-null mice. *Conclusions*. PAR1 is expressed in the mouse prostate and its activation by PAR1-TF elicits immunomodulatory effects during ethanol-DNBS-induced prostatitis. However, PAR1-TF also diminishes ethanol-DNBS-induced inflammation via a non-PAR1 mechanism by activating an as-yet unknown receptor.

## 1. Introduction

Prostatitis, resulting from both nonbacterial and bacterial causes, affects approximately 2–14% of men [1, 2]. Nonbacterial prostatitis, which accounts for 90–95% of all cases of prostatitis, presents with a diversity of inflammatory symptoms including genitourinary and pelvic pain, urinary obstruction, and ejaculatory dysfunction [3]. A uniformly effective therapy for nonbacterial prostatitis does not exist, and the pathogenesis and pathophysiology of this condition are not yet understood. To gain insight into the mechanisms of prostatitis, our lab has employed rat models of both bacterial prostatitis and nonbacterial prostatitis [4–6]. Our models of infectious prostatitis have shown that the inflammation is due to virulence factors from *Escherichia coli* and *Proteus mirabilis* [7, 8]. Although the mechanisms for *Pseudomonas aeruginosa*-induced prostatitis involve functional *las* and *rhl* quorum sensing systems for complete infection and inflammation to occur [9], the mechanisms that cause noninfectious prostatitis are essentially unknown. To explore these mechanisms, we have developed a murine hapten-induced model of prostatitis, based on our established rat model [5] to analyze nonbacterial prostatitis in the mouse. This newly developed murine model, stemming from a rodent hapten-induced model of colitis [10], enables the use of genetically altered mice to evaluate the etiology of nonbacterial prostatitis.

In our search for potential causes of noninfectious prostatitis, our attention was drawn to the presence of proteinase-activated receptors (PARs) in the prostate [11, 12], which is also known to produce PAR-activating kallikrein-related peptidases belonging to the prostate-specific antigen or PSA family [13]. Furthermore, the PARs are upregulated in pathological conditions, such as cancer of the prostate [14].

The G-protein-coupled receptor “PAR” family comprises four receptors (PARs 1–4). PARs are triggered by an unusual serine proteinase mechanism that involves the cleavage-mediated exposure of a masked amino-terminal receptor sequence [15]. This revealed sequence then acts as a “tethered ligand” to stimulate receptor signaling and induce numerous downstream effects. In particular, PAR1 is cleaved by a number of trypsin-fold serine proteinases including, among others, thrombin, plasmin, and cathepsin G [15–17]. In addition, synthetic PAR-selective activating peptides are widely used to stimulate PARs. These peptides are useful in pharmacological studies because they directly mimic the tethered ligand sequence and bypass proteolytic cleavage altogether [15, 18]. Thus, PARs can be activated by synthetic peptides to assess the impact that enzyme-mediated PAR stimulation might have in a tissue. This strategy avoids the complex effects that proteinases themselves might have, such as nontargeted substrate cleavage. Our studies specifically used the PAR1-selective activating peptide, TFLLR-NH_2_ (PAR1-TF), in the context of murine prostatitis.

PARs are known to play a prominent role in a variety of tissues including the gastrointestinal (GI) tract and the central nervous system [19, 20]. In particular, PARs 1 and 2 can be involved in both inflammatory and anti-inflammatory processes [19]. Enzymes that regulate PAR1 in such tissues range from the coagulation proteinases, such as thrombin and factor VIIa/Xa [21, 22], to the kallikrein-related peptidase family (KLKs) [23]. The prostate is recognized as an important source of proteinases and KLKs, for which the most widely recognized KLK family member, KLK3/prostate-specific antigen (PSA), is used as a prognostic indicator of prostate cancer [24, 25]. Prostate-derived KLKs are thought to promote an inflammatory process not only by cleaving kininogens, prourokinase-plasminogen activator (prouPA), and proteins of the extracellular matrix but also by activating PARs [20, 23]. Since PARs are expressed in the prostate of a number of species including humans [14, 25] and since the prostate can produce serine proteinases, we hypothesized that PAR activation may play a role in nonbacterial prostatitis.

To test this hypothesis, we developed a new hapten-induced mouse model of prostatitis to evaluate the impact of intraprostatic PAR1 activation on prostate inflammation. This model allowed us to use both wild-type and PAR1-null mice. To this end, prostatitis was induced using ethanol-DNBS in the presence or absence of the PAR-selective activating peptide, PAR1-TF, in either wild-type or PAR1-null (PAR1^−/−^) mice. Our data support an immunomodulatory anti-inflammatory role for PAR1 that involves an elevation of the anti-inflammatory cytokine IL-10. In addition, our work has revealed an unexpected non-PAR1 target for TFLLR-NH_2_ that diminishes prostatitis via a receptor that warrants further investigation.

## 2. Materials and Methods

### 2.1. Chemicals, Proteinase, and Peptides

Chemicals used in this study included anhydrous ethyl alcohol (EtOH; Commercial Alcohols) and dinitrobenzene sulfonic acid (DNBS; MP Biomedicals). PAR-activating peptides used included the synthetic PAR1 agonist peptide TFLLR-NH_2_ (PAR1-TF) and its reverse receptor-inactive peptide control RLLFT-NH_2_ (RL). All peptides were synthesized via solid-phase peptide synthesis by the Peptide Synthesis Facility at the University of Calgary. The composition and purity of all peptides were established via high performance liquid chromatography (HPLC), mass spectral analysis, and amino acid analysis.

### 2.2. Animal Model

Male C57BL/6 mice weighing approximately 21 to 28 g (4 to 8 weeks old) were obtained from the Life and Environmental Sciences Animal Resource Centre at the University of Calgary. PAR1-null C57BL/6 mice and their wild-type counterparts were obtained from an in-house breeding colony at the University of Calgary, Faculty of Medicine, from breeding pairs originally provided by Johnson & Johnson Pharmaceutical Research and Development [26]. Mice were caged in polysulfone shoebox cages with aspen chip bedding and housed at 20 ± 2°C and 40 ± 10% relative humidity, with 12 h of illumination per day. Animals were provided mouse chow and water *ad libitum*. Mice were randomly divided into groups (*n* = 4–7) and anesthetized with 4% halothane. While anaesthetized, mice were transurethrally catheterized with an ethylene oxide sterilized and lubricated PE10 polyethylene feeding tube (inner diameter 0.28 mm; outer diameter 0.61 mm) attached to a 30.5-gauge needle. Catheters were inserted into the urethra 1.5 cm from the base of the penis. All compounds were administered at a maximum volume of 40 *μ*L while the catheter was held in place. Prostatitis was induced using 10 mg/mL of DNBS dissolved in 50% EtOH and 50% 0.01 M pH 7.2 sterile phosphate-buffered saline (PBS). PAR peptides were dissolved in 25 mM HEPES buffer at pH 7.4 to a concentration of 5 mM and combined 1 : 1 with 50% EtOH and 10 mg/mL DNBS. Mice were administered the following peptides in 40 *μ*L solutions: 5 mM (100 nmol) TF or 5 mM (100 nmol) RL. The time directly following solution administration was designated as time 0 h and mice were sacrificed by cervical dislocation at intervals of 24 h, 48 h, and 72 h following transurethral catheterization. The Life and Environmental Sciences Animal Care Committee in accordance with the Canadian Council on Animal Care guidelines granted approval for this study.

### 2.3. Macroscopic Damage

The ventral prostate was aseptically removed from each mouse and visually assigned a score of 0–3, based on the severity of prostatitis. Two independent observers scored ventral prostates in a randomized order. Prostates were scored as follows: grade 0—normal appearance; grade 0.5—minor congestion; grade 1—congestion; grade 2—congestion and marked edema; and grade 3—congestion, marked edema, and hyperemia.

### 2.4. Tissue Preparation

Following macroscopic damage grading and weighing, mouse prostates were photographed and tissue was processed. Tissue was aseptically divided into three equal pieces: one piece was fixed in 10% neutral-buffered formalin for histological analyses, one piece was stored on ice for immediate myeloperoxidase (MPO) assays, and the last piece was frozen and stored at −70°C for cytokine enzyme-linked immunosorbent assays (ELISAs). Mouse prostate weights were normalized to whole body weights and expressed as a percentage.

### 2.5. Microscopic Damage

Prostate tissue was fixed as above and embedded in paraffin. Five *μ*m sections were obtained and stained with hematoxylin and eosin (H&E) according to standard protocols. The severity of prostatitis was quantified morphometrically based on three separate categories according to criteria verified by an anatomical pathologist. Scores of 0 to 3 were assigned to each category and then added together for a final score out of 9. Two independent observers scored prostate sections in a randomized order. Standardized scoring categories were as follows: (i) epithelial cell exfoliation and shedding: grade 0—normal architecture; grade 1—less than 50% of acini showed epithelial cell exfoliation; grade 2—50% of acini showed epithelial cell exfoliation; and grade 3—greater than 50% of acini showed epithelial cell exfoliation; (ii) epithelial cell necrosis: grade 0—normal architecture; grade 1—less than 50% epithelial cell necrosis; grade 2—50% epithelial cell necrosis; and grade 3—greater than 50% epithelial cell necrosis; (iii) inflammatory cell infiltrate: grade 0—normal architecture; grade 1—minor focal infiltrate; grade 2—severe focal and minor diffuse infiltrate; and grade 3—severe diffuse infiltrate. Slides were imaged on a Leica DMR microscope at 20x magnification and photomicrographs were taken using a Micropublisher 5.0 RTV digital camera (QImaging) and Volocity Acquisition v 4.4 imaging software (Improvision Inc.).

### 2.6. MPO Assay

Prostate tissue was homogenized in hexadecyltrimethyl ammonium bromide (HTAB) buffer. The homogenates were then centrifuged at 13 000 ×g for 2.5 min and the supernatants were mixed with O-dianisidine (Sigma-Aldrich) in phosphate buffer. The solution was assayed at 450 nm and activity was recorded per mg protein [27].

### 2.7. Cytokine ELISAs

Tissue examined for cytokine production was homogenized in PBS containing 2 *μ*L of protease inhibitor (Sigma-Aldrich) per mL of homogenate. The homogenates were then centrifuged at 1000 ×g for 10 min. Supernatants were assayed for IL-10, IL-1*β*, IL-6, TNF-*α* (eBiosciences), and KC/CXCL-1 (R&D Systems) according to the manufactures' instructions. Mean cytokine concentration was recorded as pg cytokine/mg protein.

### 2.8. RT-PCR

Mouse-specific cDNA primers and random hexameric cDNA primers were synthesized and gel-purified by the University of Calgary DNA Services. Whole prostates were harvested from male C57BL/6 mice treated with 0.9% (w/v) saline. Prostate tissue was homogenized in PBS and total RNA was extracted and isolated using an RNeasy kit (QIAGEN) according to the manufactures' instructions. The concentration of purified RNA was determined by measuring the A_260_ using Nanodrop 2000 technology (Thermo Scientific). Following RNA purification, 5 *μ*g of RNA was transcribed and amplified at 50°C for 50 min using a SuperScript III First-Strand Synthesis System (Invitrogen) and random hexameric cDNA primers. Once cDNA was obtained, 2 *μ*L of product was combined with primer pairs designed to amplify mouse PAR1. Primers were as follows: PAR1: 5′ primer, GCG GGC AGC CTT GGG ACA AT; 3′ primer, ATG AAG GGA GGA GGC GGC GT; *β*-actin: 5′ primer, CAC CCG CGA GCA CAG CTT CT; 3′ primer, CCT CAG GGC ATC GGA ACC GC. TopTaq DNA Polymerase (QIAGEN) was used for PCR amplification. 35 cycles were run beginning with a 92°C denaturing step for 30 sec, followed by a 54°C annealing step for 30 sec, and lastly a 72°C extension step for 1 min with a final 72°C extension step for 1 min. PCR products were separated by 2.5% (w/v) agarose gel electrophoresis and visualized with SYBR Safe (Invitrogen).

### 2.9. Immunohistochemistry

This study adapted and modified immunohistochemistry methods previously described using rabbit polyclonal anti-PAR1 antisera [28, 29]. Formalin-fixed, paraffin-embedded sections 5 *μ*m in thickness of mouse prostate tissue (treated with 0.9% saline) were deparaffinized and hydrated using decreasing concentrations of ethanol. Sections were boiled in 0.01 M pH 6.0 citrate buffer for 10 min and endogenous peroxidase activity was blocked by incubating sections in 0.3% H_2_O_2_ (Sigma-Aldrich) for 20 min. Nonspecific binding was prevented by preincubating sections with 10% normal goat serum/0.5% Triton X-100 for 1 h at room temperature. To detect PAR1 immunoreactivity, sections were incubated overnight at room temperature with a rabbit polyclonal antibody raised against a synthetic mouse PAR1 peptide. This peptide spans the thrombin cleavage-activation site (//), including the “tethered ligand” sequence (underlined) (YATPNPR//SFFLRNPSEDGGC: 1/500), and was diluted in PBS with 10% normal goat serum. Immunolabeling of the primary PAR1 antibody was detected using a secondary biotinylated goat anti-rabbit antibody followed by avidin-biotin peroxidase complexes (Vector Laboratories) for 1 h at room temperature. Peroxidase activity was verified using 0.5 mg/mL 3,3′-diaminobenzidine tetrachloride (Sigma-Aldrich) in 0.05 M pH 7.6 Tris-HCl buffer containing 0.03% H_2_O_2_. Sections were then counterstained with hematoxylin (Vector Laboratories) for 30 min, dehydrated, and mounted. All washes were performed for 15 min with 0.01 M pH 7.2 PBS. Slides were imaged on a Leica DMR microscope at 100x magnification and photomicrographs were taken using a Micropublisher 5.0 RTV digital camera (QImaging) and Volocity Acquisition v 4.4 imaging software (Improvision Inc.).

### 2.10. Statistical Analysis

Group data are presented as means ± standard error of the mean (SEM). Figures and statistical examination were compiled using GraphPad Prism v 5.00 software (Graphpad Software). Macroscopic and microscopic damage scores were analyzed by a Mann-Whitney *U* test or a Kruskal-Wallis one-way analysis of variance (ANOVA) followed by Dunn's multiple comparison test. MPO assays and cytokine ELISAs were analyzed by Student *t*-test or a one-way ANOVA followed by a Tukey's multiple comparison test. A *P* value of <0.05 was considered statistically significant.

## 3. Results

### 3.1. Ethanol and DNBS Induce Nonbacterial Prostatitis in the Mouse

**Figure 1:**
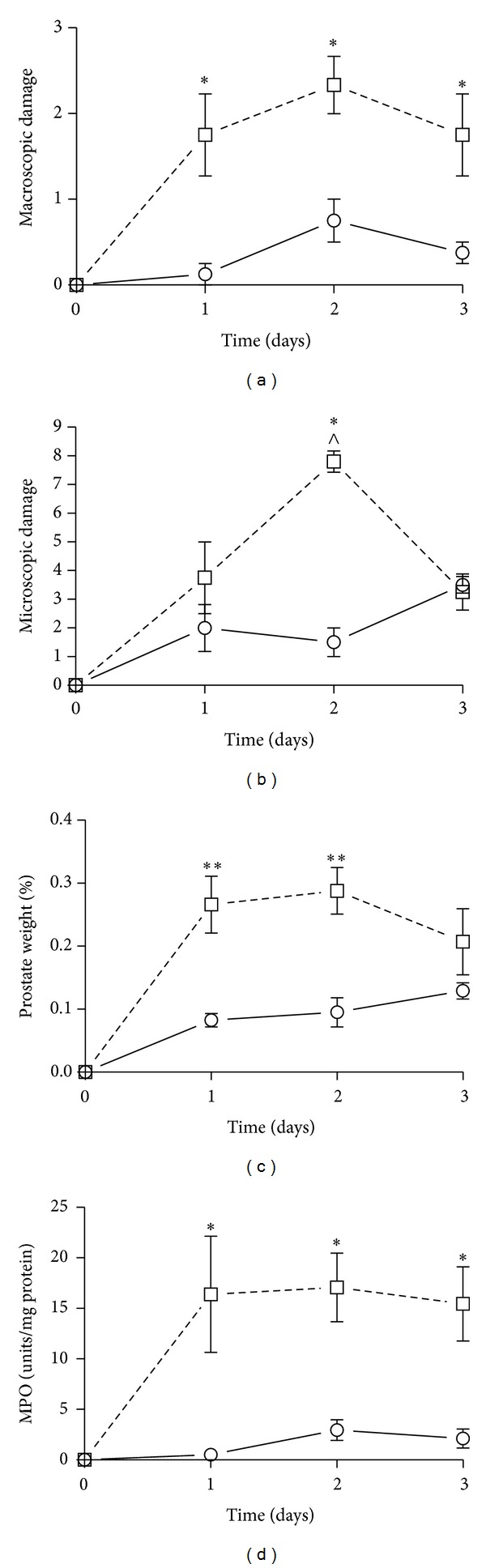
Ethanol + dinitrobenzene sulfonic acid induces inflammatory changes in wild-type mice 2 days posttreatment. Prostates were treated with sterile saline (circles) or ethanol + dinitrobenzene sulfonic acid (squares) for 1, 2, or 3 days starting on day 0. The inflammatory response was measured by monitoring (a) macroscopic prostate damage, (b) microscopic prostate damage, (c) percent prostate weight, and (d) myeloperoxidase (MPO) activity. Data are mean ± SEM of *n* = 4–6 per point. *P < 0.05, **P < 0.01 for EtOH + DNBS versus saline; ^∧^ *P* < 0.05 for 2-day EtOH + DNBS versus 3-day EtOH + DNBS.

**Figure 2:**
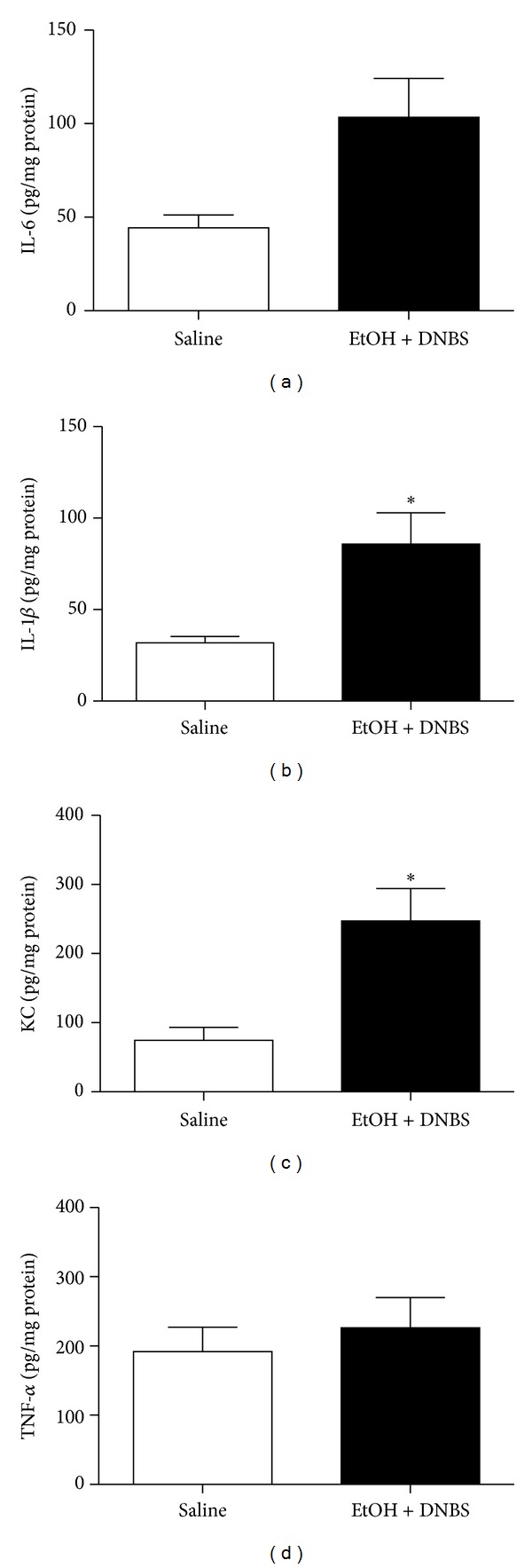
Cytokine production is elevated when ethanol + dinitrobenzene sulfonic acid is administered in wild-type mice. Prostates were treated with sterile saline (saline) or ethanol + dinitrobenzene sulfonic acid (EtOH + DNBS) for 2 days. The inflammatory response was measured by monitoring (a) interleukin-6 (IL-6), (b) interleukin-1*β* (IL-1*β*), (c) keratinocyte-derived cytokine (KC), and (d) tumor necrosis factor-*α* (TNF-*α*). Data are mean ± SEM of *n* = 4–6 per group. *P < 0.05 for EtOH + DNBS versus saline.

We first established a hapten-based murine model of acute nonbacterial prostatitis based on our previously published rat model [5]. In keeping with the rat data, administration of ethanol-DNBS induced a prominent inflammatory reaction in the mouse prostate that, over a 3-day time period, was maximal at day 2 (Figure 1). At that time point, ethanol-DNBS-treated mouse prostates showed all of the hallmarks of inflammation including increased macroscopic and microscopic damage scores, increased prostatic weight gain, and elevated MPO activity (Figures 1(a), 1(b), 1(c), and 1(d)). Based on these data, a 2-day ethanol-DNBS time course was used to model nonbacterial prostatitis throughout the remainder of this study. These ethanol-DNBS-treated prostates also showed significantly elevated IL-1*β* and KC inflammatory cytokine production (Figures 2(b) and 2(c)). Histologically, the ethanol-DNBS-treated prostate tissue showed widespread leukocyte infiltration, hemorrhage, extensive destruction of the prostatic acini and epithelium, and epithelial cell necrosis (Figure 6(b)). Conversely, saline controls showed a healthy and undamaged prostatic architecture (Figure 6(a)).

### 3.2. PAR1 Is Expressed in the Mouse Prostate

**Figure 3:**
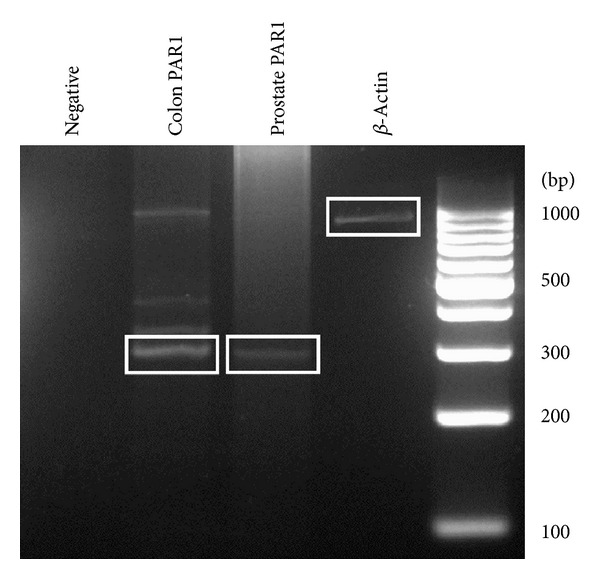
PAR1 mRNA is expressed in the wild-type mouse prostate. Prostates were treated with sterile saline and sacrificed 2 days posttreatment. Primers specific for mouse PAR1 were used to amplify cDNA that was reverse transcribed from homogenized mouse prostate tissue. RT-PCR lanes from left to right are as follows: primers without cDNA (negative), PAR1 colon cDNA (colon PAR1), PAR1 prostate cDNA (prostate PAR1), and *β*-actin (*β*-actin). A 100 bp DNA ladder was used.

**Figure 4:**
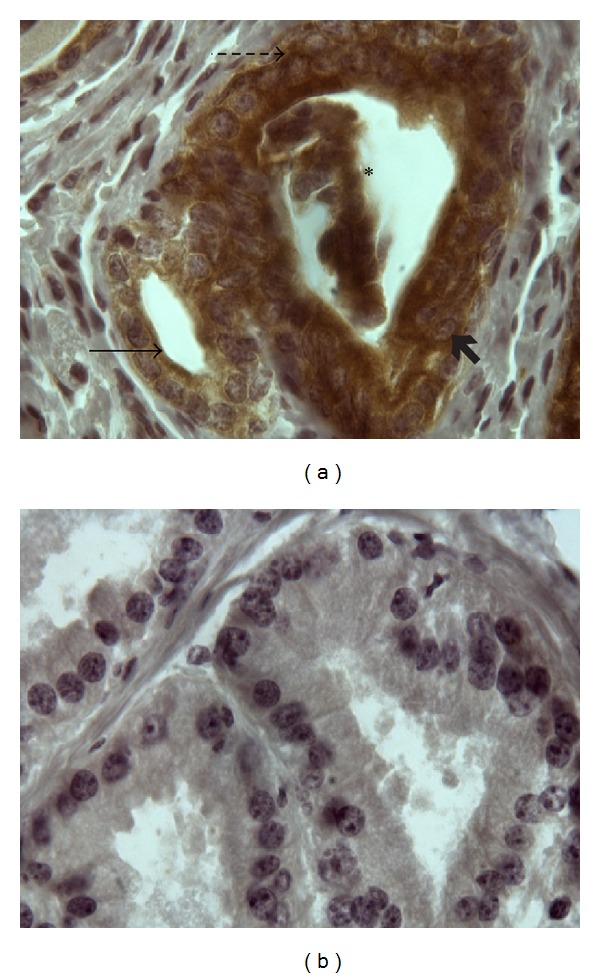
PAR1 is expressed in the wild-type mouse prostate. Prostates were treated with sterile saline and sacrificed 2 days posttreatment. PAR1 expression was localized via immunohistochemistry and was as follows. (a) Positive PAR1 staining throughout the apical acini epithelium (arrow), prostatic acini lumen (*), basal acini epithelium (dashed arrow), and prostatic epithelial cells (arrowhead) in wild-type mice. (b) Negative PAR1 staining in PAR1 knockout (PAR1^−/−^) mice. Sections were counterstained with hematoxylin; original magnification 100x.

A PAR1 PCR product of the expected size (296 bp) was detected in mouse prostate tissue (Figure 3). The PAR1 PCR product was identical to the one verified by us in mouse colon tissue. The cellular localization of PAR1 was determined using immunohistochemistry. Prostatic PAR1 was localized prominently throughout the apical acini epithelium and acini lumen as well as throughout the basal acini epithelium and prostatic epithelial cells. There was specific staining both at the plasma membrane of cells (Figure 4(a): arrows) and diffuse intracellular reactivity. No immunoreactivity was observed using a nonimmune rabbit serum control, and the anti-PAR1 antiserum did not yield a signal in PAR1-null mice (Figure 4(b)). In other tissues such as mouse liver, PAR1 immunoreactivity was also detected with our antiserum, as reported previously [30] (data not shown).

### 3.3. The PAR1-Activating Peptide, TFLLR-NH_2_ (PAR1-TF), Has Anti-Inflammatory Effects in the Context of Ethanol-DNBS-Mediated Prostatitis

**Figure 5:**
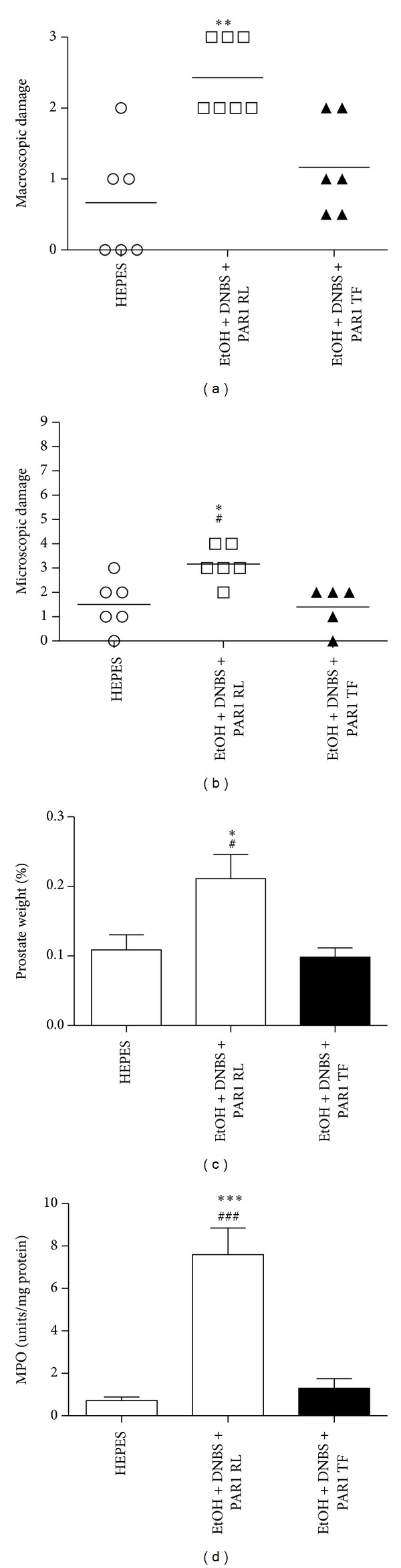
Instillation of the PAR1 activating peptide TFLLR-NH_2_ diminishes ethanol- + dinitrobenzene sulfonic acid-induced prostatitis in wild-type mice. Prostates were treated with HEPES vehicle (HEPES), ethanol + dinitrobenzene sulfonic acid + PAR1 reverse peptide RLLFT-NH_2_ (EtOH + DNBS + PAR1 RL), or ethanol + dinitrobenzene sulfonic acid + PAR1 activating peptide TFLLR-NH_2_ (EtOH + DNBS + PAR-1 TF) for 2 days. The inflammatory response was measured by monitoring (a) macroscopic prostate damage, (b) microscopic prostate damage, (c) percent prostate weight, and (d) myeloperoxidase (MPO) activity. Data are mean ± SEM of *n* = 5-6 per group. *P < 0.05, **P < 0.01, and ***P < 0.001 for EtOH + DNBS + PAR1 RL versus HEPES; ^#^ *P* < 0.05, ^###^ *P* < 0.001 for EtOH + DNBS + PAR1 RL versus EtOH + DNBS + PAR1 TF.

**Figure 6:**
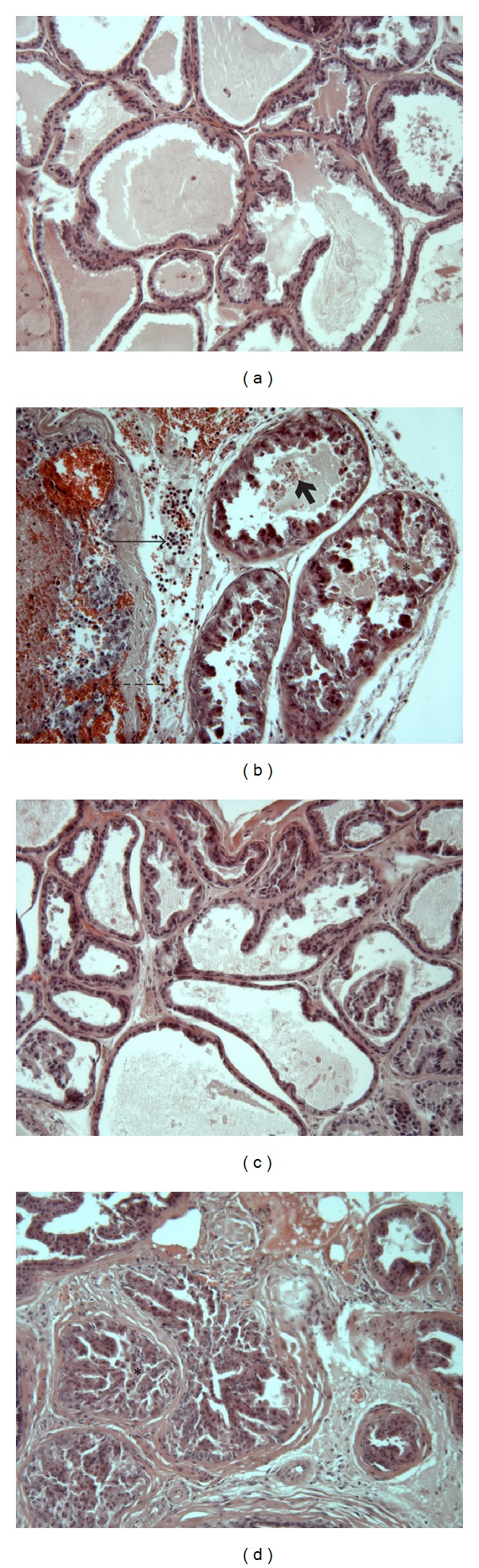
Instillation of the PAR1 activating peptide TFLLR-NH_2_ diminishes ethanol- (EtOH-) + dinitrobenzene sulfonic acid- (DNBS-) induced microscopic damage in wild-type mice. Prostates were treated for 2 days with (a) sterile saline, (b) EtOH + DNBS (arrow indicates leukocyte recruitment, dashed arrow indicates hemorrhage, asterisk indicates acini destruction, and arrowhead indicates epithelial cell necrosis), (c) EtOH + DNBS + PAR1 activating peptide TFLLR-NH_2_ (TF), and (d) EtOH + DNBS + PAR1 reverse peptide RLLFT-NH_2_ (RL) (asterisk indicates acini destruction). All sections were stained with hematoxylin and eosin (H&E); original magnification 20x.

As outlined above, administration of ethanol-DNBS into the mouse prostate induced pronounced inflammation, as measured by macroscopic and microscopic damage, prostatic weight gain, MPO activity, and cytokine production (Figures 1 and 2). On its own, the PAR1-activating peptide did not affect the indices of prostate inflammation (data not shown). However, when coadministered with ethanol-DNBS, PAR1-TF markedly reduced the ensuing prostatitis, relative to animals treated with ethanol-DNBS alone. These PAR1-TF-treated prostates showed significantly diminished microscopic damage, weights, and MPO activity, when compared to control prostates treated with the PAR1-inactive reverse peptide (ethanol-DNBS-PAR1-RL) (Figures 5(b), 5(c), and 5(d)). Histological examination of prostate tissue treated with ethanol-DNBS in combination with PAR1-TF showed very little leukocyte recruitment throughout the prostatic stroma or acini, and the prostatic epithelium was undamaged (Figure 6(c)). Overall, sections from mice treated with ethanol-DNBS and PAR1-TF were comparable to noninflamed sections treated with saline. In contrast, tissue treated with the reverse-sequence, receptor-inactive PAR1-derived peptide (PAR1-RL) along with ethanol-DNBS displayed destruction of the prostatic epithelium and acini, as in the inflamed sections treated with ethanol-DNBS alone (Figures 6(b) and 6(d)).

### 3.4. PAR1 TFLLR-NH_2_ (PAR1-TF) Directly Upregulates IL-10 Production in the Context of Ethanol-DNBS-Induced Prostatitis

**Figure 7:**
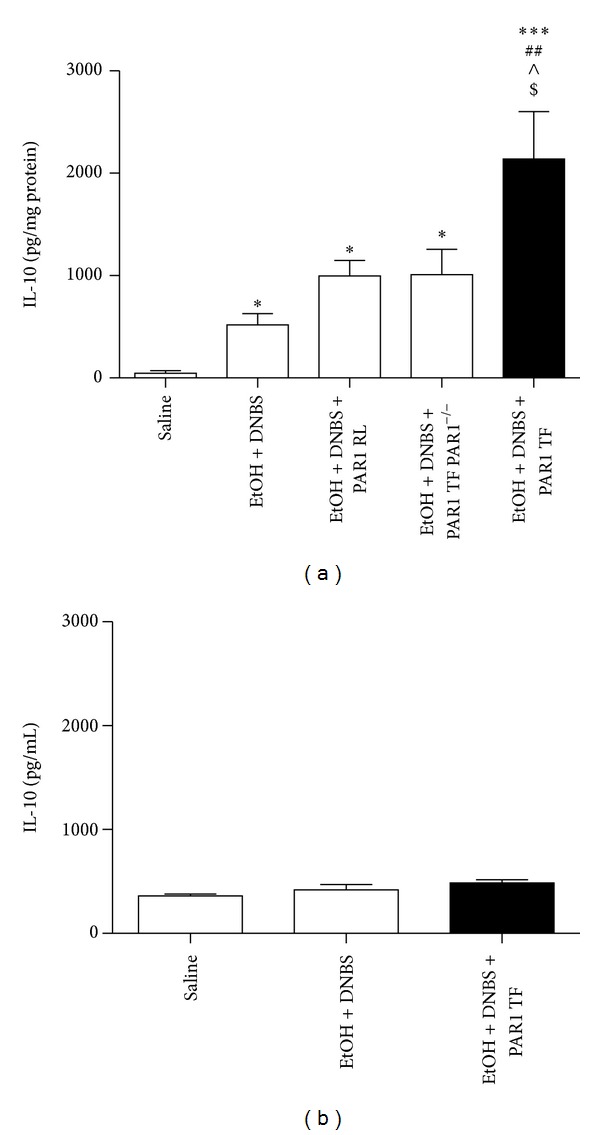
Instillation of the PAR1 activating peptide TFLLR-NH_2_ significantly increases IL-10 concentration in the inflamed prostates of wild-type but not PAR1^−/−^ mice and does not change IL-10 levels in serum. Prostates were treated with sterile saline in wild-type mice (saline), ethanol + dinitrobenzene sulfonic acid in wild-type mice (EtOH + DNBS), ethanol + dinitrobenzene sulfonic acid + PAR1 reverse peptide RLLFT-NH_2_ in wild-type mice (EtOH + DNBS + PAR1 RL), ethanol + dinitrobenzene sulfonic acid + PAR1 activating peptide TFLLR-NH_2_ in PAR1 knockout mice (EtOH + DNBS + PAR1 TF PAR1^−/−^), or ethanol + dinitrobenzene sulfonic acid + PAR1 activating peptide TFLLR-NH_2_ in wild-type mice (EtOH + DNBS + PAR1 TF) for 2 days. The inflammatory response was measured by monitoring (a) prostatic interleukin-10 (IL-10), and (b) serum IL-10. Data are mean ± SEM of *n* = 4–7 per group. *P < 0.05, ***P < 0.001 for treatment groups versus saline; ^##^ *P* < 0.01 for EtOH + DNBS + PAR1 TF versus EtOH + DNBS; ^∧^ *P* < 0.05 for EtOH + DNBS + PAR1 TF versus EtOH + DNBS + PAR1 RL; ^$^ *P* < 0.05 for EtOH + DNBS + PAR1 TF versus EtOH + DNBS + PAR1 TF PAR1^−/−^.

In accordance with the anti-inflammatory effects of PAR1-TF shown in Figure 5, we found that TFLLR-NH_2_ upregulated production of the anti-inflammatory cytokine, IL-10. Treatment with ethanol-DNBS alone led to a small increase in prostatic IL-10 production versus saline instillation (Figure 7(a)). This increase was slightly augmented by the addition of the PAR1-inactive reverse peptide to the ethanol-DNBS solution (EtOH + DNBS + PAR1-RL: third histogram from *y*-axis, Figure 7(a)). However, the impact of the receptor-active peptide, PAR1-TF, on IL-10 production when administered along with ethanol-DNBS in PAR1-null mice was not different from the effect of the reverse-sequence receptor-inactive peptide in wild-type mice (third versus fourth histograms from *y*-axis, Figure 7(a)). Strikingly, in the PAR1 wild-type mice, coadministration of receptor-active PAR1-TF to ethanol-DNBS-treated wild-type prostates led to a significantly increased IL-10 production versus all other treatment groups (Figure 7(a), fifth histogram from *y*-axis). As already pointed out, this marked elevation due to PAR1-TF was not observed in PAR1-null animals and was not observed in wild-type mice treated with ethanol-DNBS along with the reverse-sequence PAR1-inactive peptide, PAR1-RL (Figure 7(a), third and fourth histograms from *y*-axis). Significant differences in the levels of IL-10 in the serum (as opposed to the tissue extracts) were not found between the different treatment groups, suggesting that PAR1-TF had no effect on systemic IL-10 levels (Figure 7(b)).

### 3.5. The PAR1-Activating Peptide, TFLLR-NH_2_ (PAR1-TF), Is Anti-Inflammatory in PAR1-Deficient Mice in the Context of Ethanol-DNBS-Induced Prostatitis

**Figure 8:**
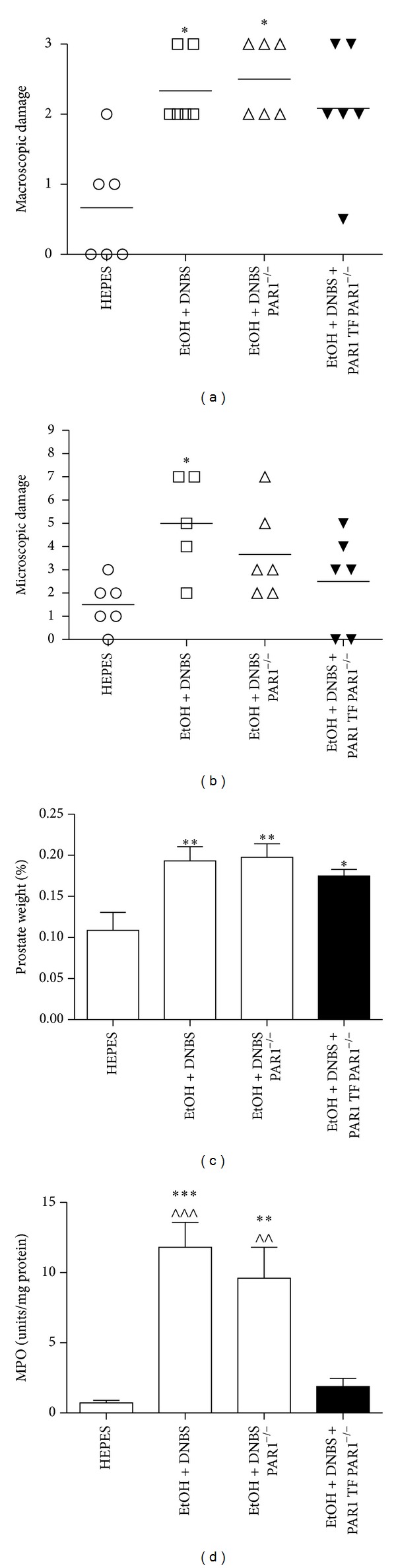
Administration of the PAR1 activating peptide TFLLR-NH_2_ elicits non-PAR1-mediated effects in the mouse prostate. Prostates were treated with HEPES vehicle in wild-type mice (HEPES), ethanol + dinitrobenzene sulfonic acid in wild-type mice (EtOH + DNBS), ethanol + dinitrobenzene sulfonic acid in PAR1 knockout mice (EtOH + DNBS PAR1^−/−^), or ethanol + dinitrobenzene sulfonic acid + PAR1 activating peptide TFLLR-NH_2_ in PAR1 knockout mice (EtOH + DNBS + PAR1 TF PAR1^−/−^) for 2 days. The inflammatory response was measured by monitoring (a) macroscopic prostate damage, (b) microscopic prostate damage, (c) percent prostate weight, and (d) myeloperoxidase (MPO) activity. Data are mean ± SEM of *n* = 5-6 per group. *P < 0.05, **P < 0.01, and ***P < 0.001 for treatment groups versus HEPES; ^∧∧^ *P* < 0.01, ^∧∧∧^ *P* < 0.001 for treatment groups versus EtOH + DNBS + PAR-1 TF PAR1^−/−^.

The observation of an anti-inflammatory action of the receptor-selective PAR1-TF agonist, with an absence of an anti-inflammatory action of the reverse-sequence PAR-inactive peptide PAR1-RL, strongly pointed pharmacologically to an anti-inflammatory role for PAR1. We wished to evaluate this putative anti-inflammatory role for PAR1 further by analyzing the impact of the peptides in PAR1-null mice. The intraprostatic administration of ethanol-DNBS induced an inflammatory reaction in PAR1-null mice comparable to that observed in the prostates of wild-type mice. Specifically, in PAR1-null mice, as in the wild-type animals, ethanol-DNBS significantly increased macroscopic damage scores, prostatic weight gain, and MPO activity, when compared to HEPES controls (Figures 8(a), 8(c), and 8(d)). To our surprise, in the PAR1-null mice, the coadministration of the PAR1-activating peptide along with ethanol-DNBS substantially diminished most of the indices of inflammation. Histological examination of these PAR1-null mice treated with ethanol-DNBS along with PAR1-TF revealed very little leukocyte infiltrate, healthy prostatic acinus, stroma, and epithelium (data not shown) and markedly attenuated histological tissue damage and MPO activity, similar to the effect of the PAR1-activating peptide in the wild-type mice (Figure 8(d)). However, the coadministration of PAR1-TF with ethanol-DNBS did not diminish prostatic weights in the PAR1-null mice (Figure 8(c)). Thus, in this respect, the PAR1-active peptide was not active in the PAR1-null mice. These data suggest both PAR1-dependent and surprisingly PAR1-independent anti-inflammatory effects of the PAR1-activating peptide in the murine prostatitis model.

## 4. Discussion

We found that the murine ethanol-DNBS prostatitis model accurately reflected our previous work with the rat model [5], with a maximum of inflammatory indices observed at day 2. It is of interest to note that microscopic damage scores decreased dramatically from day 2 to day 3 posttreatment (Figure 1(b)). This may indicate that inflammatory resolution was occurring on a microscopic level between day 2 and day 3 posttreatment. Nonetheless, day 2 showed maximal microscopic prostate damage and was used throughout the study as the experimental time point. In addition to causing an increase in all of the indices of inflammation in the tissue, the ethanol-DNBS-induced prostatitis resulted in an elevation of the tissue proinflammatory cytokines IL-6, IL-1*β*, and KC but not TNF-*α* (Figure 2). Histologically, it can be suggested that the fixation process may have altered the morphology of the prostatic acini and/or epithelium. Saline-treated controls, however, were processed in an identical manner and did not show any destruction of the acini or epithelium, arguing against a fixation-induced artifact for the histology specimens.

Our main focus with the use of this murine model was to evaluate a potential role for PAR1 in murine noninfectious prostatitis. Clearly, PAR1 is present in the mouse prostate, as indicated by our RT-PCR and immunohistochemical data. These findings are in keeping with the detection of PARs in human normal and cancerous prostatic epithelial cells *in vitro* [25, 31] and in cancerous prostate tissue *in vivo* [14]. We observed that PAR1 in the mouse prostate was localized to the apical acini epithelium and lumen and expressed to a lesser degree along the basal acini epithelium and epithelial cells. Further, we found intracellular PAR1 immunoreactivity as detected in other works. Thus, PAR1 is present in the mouse prostate and can in principle readily play a role in murine prostatic function. Of note, although PAR3 and PAR4 are both expressed in human prostate tissue, data regarding PAR3 and PAR4 expression in mouse prostate tissue is limited. Based on our PAR1 results, we hypothesize that PAR3 and PAR4 are expressed in the mouse prostate. The receptor-selective PAR1-activating peptide, however, unequivocally does not activate PAR3 or PAR4, as its amino acid sequence is specific for the tethered ligand of PAR1. It is possible that upregulated thrombin or other enzymes in PAR1-null mice may activate PAR3 and PAR4, thereby surrogating PAR1 function. Further investigation is required to completely elucidate the involvement of PAR3 and PAR4 in PAR1-null mice.

In this study, ethanol-DNBS and the PAR1-activating peptide were administered concurrently into the mouse prostate. Thus, the impact of PAR1 was on acute injury and our results indicated that the PAR1-activating peptide dampened the inflammatory response triggered by ethanol-DNBS. In particular, the main finding of our work dealing with PAR1 in our prostatitis model was that the PAR1-activating peptide, TFLLR-NH_2_, could cause both a PAR1-dependent and a PAR1-independent anti-inflammatory effect. In this regard, the use of both PAR1-null mice and peptide structure-activity information with a PAR1-active versus a PAR1-inactive peptide was essential to identify which effects of the PAR1-activating peptide were due to PAR1 itself and which effects were PAR1 independent. Our data show that the reverse-sequence PAR1 peptide, RLLFT-NH_2_, was not able to generate an anti-inflammatory effect in either wild-type or PAR1-null mice, indicating that some of the effects of the PAR1-TF peptide (TFLLR-NH_2_) were PAR1 dependent. Further, the PAR1-active peptide, TFLLR-NH_2_ was not able to elevate IL-10 or inhibit tissue edema (reflected by increased prostate weight) in the PAR1-null mice. Thus, at least a portion of the anti-inflammatory action of PAR1-TF related to IL-10 cytokine production and swelling was PAR1-dependent. Because IL-10 is a pleiotrophic cytokine with both anti-inflammatory and immunosuppressive properties, these data support a direct PAR1-mediated anti-inflammatory role that has also been described in previous reports. For example, PAR1 agonists have promoted the release of IL-10 while inhibiting the production of TNF-*α* and IL-6 in mouse microglial cells [32]. In addition PAR1 agonists increase IL-10 production in both resting and activated peripheral blood mononuclear cells [33]. Together, these data support the hypothesis that PAR1 activation may mediate anti-inflammatory effects in nonbacterial prostatitis.

To our surprise, however, the receptor-active peptide, TFLLR-NH_2_, was able to diminish many of the indices of ethanol-DNBS-triggered inflammation in *both* the PAR1-null and PAR1-wild-type mice. Thus, our work, like other studies, demonstrates the caution with which the PAR-activating peptides must be used to elucidate the potential impact of PAR activation in tissues. Previous studies have indicated that non-PAR-mediated effects can occur with other PAR-selective peptide agonists. For example, it has been demonstrated that PAR1 and PAR2 agonists may cause mast cell mediator release via non-PAR1 and non-PAR2 mechanisms [34]. Further, a relatively potent and selective PAR2-activating peptide, trans-cinnamoyl-LIGRL-NH_2_, can have off-target non-PAR2 effects in some vascular preparations but not others [35]. Thus, the use of the PAR1-null mice was a key for us to sort out the PAR1-dependent versus PAR1-independent actions of TFLLR-NH_2_. The data obtained with the PAR1-null mice revealed a novel anti-inflammatory non-PAR1 effect of TFLLR-NH_2_ (but not its reverse-sequence peptide) in a mouse model of nonbacterial prostatitis. The receptor responsible for these PAR1-independent anti-inflammatory effects of TFLLR-NH_2_ remains to be determined.

## 5. Conclusions 

Using a newly developed murine model of ethanol/DNBS-mediated prostatitis, we were able to evaluate a potential role for proteinase-activated receptor-1 (PAR1) in modulating the inflammatory response. Employing a PAR1-activating peptide, TFLLR-NH_2_, and its receptor-inactive reverse-sequence peptide, RLLFT-NH_2_, along with experiments done in PAR1-null mice, we established not only that (1) PAR1 can play an anti-inflammatory role in noninfectious prostatitis by elevating the anti-inflammatory cytokine IL-10 and diminishing swelling but also that (2) the PAR1-activating peptide (but not its reverse-sequence agonist) can, via a non-PAR1 mechanism, exhibit substantial anti-inflammatory actions. Thus, peptidomimetic agonists based on the TFLLR-NH_2_ structure may prove to be of value as therapeutic agents for the treatment of noninfectious prostatitis. These agents would diminish prostatitis by a “dual” receptor mechanism involving both PAR1 and an as-yet unidentified receptor.
